# Effects of half-dose and full-dose GnRH antagonists on IVF-ET outcomes: a retrospective study

**DOI:** 10.1186/s12884-021-04176-8

**Published:** 2021-10-27

**Authors:** Yingge Zhao, Fang Lian, Shan Xiang, Yi Yu, Conghui Pang, Yue Qiu

**Affiliations:** 1 Shandong University of Traditional Chinese Medicine, Jinan, China; 2 Affiliated Hospital of Shandong University of Traditional Chinese Medicine, Jinan, China

**Keywords:** GnRH antagonists, IVF-ET

## Abstract

**Background:**

Gonadotropin-releasing hormone antagonist(GnRH-ant) has been shown to have a negative effect on endometrial receptivity. Therefore, the use of lower doses of GnRH-ant during controlled ovarian stimulation (COS) may improve endometrial receptivity and clinical pregnancy rate. However, the GnRH-ant dose is relatively flexible and there is no fixed requirement for guidance. In this retrospective study, we determined the effects of half-dose and full-dose GnRH-ant on IVF-ET outcomes.

**Methods:**

Of the 316 cycles in the 314 patients analyzed in this study, 149 received GnRH-ant half-dose (Group1), while 167 received GnRH-ant full-dose (Group2). The groups were further classified based on age and BMI. Age subgroups, were divided as age ≤ 35(subgroup A) and age > 35(subgroup B): 180 cycles in subgroup A (107 cycles in subgroup A1,73 cycles in subgroup A2), 136 cycles in subgroup B (42 cycles in subgroup B1,94 cycles in subgroupB2). The subgroups based on BMI were divided as BMI < 25 (subgroup C)and BMI ≥ 25 (subgroup D):208 cycles in subgroup C (94 cycles in subgroup C1,114 cycles in subgroup C2), 108 cycles in subgroup D (55 cycles in subgroup D1,53 cycles in subgroup D2).

**Results:**

The number of fertilized oocytes, superior-quality embryos, clinical pregnancy rate, and live birth rate differed significantly between the two groups. However, the number of retrieved oocytes and available embryos were significantly higher in Group 1 than Group 2 (8.17 ± 4.10 vs. 7.07 ± 4.05, 2.96 ± 2.03 vs. 2.52 ± 1.62, respectively,*p*<0.05). Differences between the age subgroups were not statistically significant. However, in the subgroups based on BMI, the fertilized oocytes, available embryos, the number of superior-quality embryos, and the live birth rate differed significantly between the four subgroups. The number of retrieved oocytes was higher in subgroup C1 than in subgroup C2 (8.24 ± 4.04 vs. 6.83 ± 3.92,*p* < 0.05), In addition, the clinical pregnancy rate was slightly higher in subgroup D1 than in subgroup D2(45.45 vs. 24.53%, *P* < 0.05).

**Conclusions:**

The results showed that half-dose GnRH-ant was as effective as full-dose GnRH-ant for most patients. Moreover, half-dose GnRH-ant may be more suitable in patients with BMI greater than or equal to 25. The findings of this study need to be validated in a large sample RCT.

**Trial registration:**

Retrospectively registered.

## Background

In recent years, the gonadotropin-releasing hormone antagonist (GnRH-ant) protocol has gained clinical recognition due to the absence of early pituitary inhibition, lower doses, short duration of administration, lack of flare-ups in the initial stages of administration, and lower risk of ovarian hyperstimulation syndrome (OHSS) [1]. The GnRH-ant protocol is more in line with the physiological process. However, many studies have shown that the GnRH-ant protocol affects endometrial receptivity and interferes with the embryo implantation window [2]. Therefore, reducing the GnRH antagonist dose may positively affect the rate of embryo implantation. In general, the conventional dose of daily GnRH-ant 0.25 mg is used [3]. However, daily doses of 0.125 mg GnRH-ant dose have been used [4]. The dosage is relatively flexible. Therefore, we compared the pregnancy rate and live birth rate of ovulation induction patients with different dosages of GnRH antagonists and explored the effect of the dosage of antagonists on IVF-ET outcomes.

## Methods

### Patients

In this retrospective study, we obtained data of patients who received GnRH-ant protocol for IVF-ET/ ICSI from January 2015 to December 2020 at the Reproductive Medical Center of Affiliated Hospital of Shandong University of traditional Chinese medicine. Approval to conduct this study was sought from the institutional review board.

### Inclusion/exclusion criteria

The inclusion criteria were as follows: (a) both husband and wife had treatment indications for IVF / ICSI, (b) signed informed consent [5],(c) basal serum FSH < 15 IU/L. The exclusion criteria were: (a) chromosomal aberrations in the mother or father, (b) thyroid disease, endometriosis, or immune disease.

### Groups according to different GnRH-ant protocols

Patients were divided into two groups depending on the dose of Cetrorelix used: Group 1 (*n* = 149) initially received 0.125 mg of Cetrorelix daily. The patients in Group 1 were further divided into four subgroups depending on age (subgroup A,age ≤ 35; subgroup B,age > 35) and BMI (subgroup C,BMI < 25; subgroup D,BMI ≥ 25). In contrast, Group 2 (*n* = 167) initially received 0.25 mg of Cetrorelix daily and was further subgrouped into four based on the above categories. See the attached Tables 1 and 2 for a detailed description. Only the cetrorelix dosage was varied during COS between the two groups.

**Table 1:** Subgroup classifications of age included

Subgroup	Subgroup A1	Subgroup A2	Subgroup B1	Subgroup B2
The dose of Cetrorelix per day	0.125 mg	0.25 mg	0.125 mg	0.25 mg
Age (years)	age ≤ 35	age ≤ 35	age>35	age>35

**Table 2:** Subgroup classifications of BMI included

Subgroup	Subgroup C1	Subgroup C2	Subgroup D1	Subgroup D2
The dose of Cetrorelix per day	0.125 mg	0.25 mg	0.125 mg	0.25 mg
Body mass index	BMI<25	BMI<25	BMI ≥ 25	BMI ≥ 25

### IVF-ET procedure

The ovarian stimulation was initiated on menstrual cycle day 2 or 3 by administering recombinant FSH (Gonal-F, Merck- Serono SA, Switzerland). The initial gonadotropin dose, which typically ranged from 150 to 300 u per day was experientially determined by doctors depending on age, antral follicle count (AFC), basal FSH, E2 levels, and body mass index (BMI). The dose was adjusted every 2–3 days of stimulation depending on the ovarian response evidenced by E2 levels, LH levels, and follicular growth detected on ultrasound examination. When the diameter of the follicle was ≥14 mm, or the dominant follicle diameter was more than 12 mm and serum E2 > 300 pg/ml, the patients received the GnRH-ant Cetrorelix acetate(Cetrotide, Merck-Serono SA, Switzerland) at a dose of 0.125 mg/d or 0.25 mg/d. Finally, 10,000 IU of human chorionic gonadotropin (hCG; Lizhu, Zhuhai, China) or 250 IU of recombinant human choriogonadotro-pinalfa solution (OVIDREL;Merck-Serono SA, Switzerland) was administered when two follicles reached a mean diameter of 17 mm or one follicle reached a mean diameter of 18 mm. Oocyte retrieval was performed 35–36 h after hCG or OVIDREL injection under transvaginal ultrasound-guided single-lumen needle aspiration. Intracytoplasmic sperm injection (ICSI) was performed only in case of severe male infertility. Oocyte culture, insemination, embryo transfer, and cryopreservation were done as previously described [6]. Embryo transfer was conducted on day three after oocyte retrieval. All patients received embryo transfer on day 3, except in the following cases: (a) serum estrogen > 5007 pg/mL on the trigger day, (b) retrieval of more than 15 oocytes, (c) presence of uterine or endometrial abnormalities such as endometriosis, uterine myoma, endometrial polyps, or intrauterine adhesion, (d) an initial increase of progesterone over 1.5 ng/mL before the trigger day [7], or (e) the patient declined fresh embryo transfer. A maximum of two embryos were transferred. During the luteal phase, 20 mg progesterone injection was injected twice a day from the first day after oocyte retrieval. Clinical pregnancy was determined by visualizing a gestational sac on ultrasound at 6 weeks of gestation. Live birth was defined as having at least one live baby born after 28 weeks of gestation. Live birth rate referred to the percentage of women who gave live birth in a fresh transplant cycle [8].

### Statistical analysis

Data were analyzed using SPSS version 18.0 (IBM). Frequency for qualitative variables and the mean and standard deviation for quantitative variables were calculated. The chi-square test, Fisher’s exact test and the Student’s t-test were used to determine differences between independent samples. Statistical significance was defined as *p* < 0.05.

## Results

Table 3 presents the demographic characteristics of both groups. There were no significant differences in the patient’s age, BMI, Years of infertility, and basal FSH and LH between the two groups.

**Table 3:** Comparison of demographic characteristics between the two groups

Groups	Group1 (*n* = 149)	Group2 (*n* = 167)	*P*
Age (years)	32.56 ± 3.54	33.22 ± 3.55	0.104
Years of infertility	3.31 ± 2.46	3.54 ± 3.53	0.498
Body mass index	24.16 ± 4.38	23.94 ± 4.37	0.658
bFSH/bLH	1.80 ± 1.14	2.03 ± 1.76	0.195

*Data expressed as mean ± SD, or number (percentage)

Tables 4 and 5 presents the demographic characteristics of the four subgroups. There were no significant differences in the patient’s age, BMI, Years of infertility, and basal FSH and LH between the four subgroups.

**Table 4:** Comparison of demographic characteristics between subgroups

Subgroup	Subgroup A1(*n* = 107)	Subgroup A2(*n* = 73)	Subgroup B1(*n* = 42)	Subgroup B2(*n* = 94)	*P*
Years of infertility	2.96 ± 1.82	3.29 ± 1.97	4.14 ± 3.48	3.79 ± 4.39	>0.05
Body mass index	23.77 ± 4.79	22.75 ± 5.48	24.10 ± 3.24	23.85 ± 5.81	>0.05
bFSH/bLH	1.83 ± 1.14	2.06 ± 2.01	1.80 ± 1.14	1.93 ± 1.53	>0.05

*Subgroup A1, Subgroup A2 basic data *p* values were:0.257, 0.187, 0.319

*Subgroup B1, Subgroup B2 basic data *p* values were:0.644, 0.793, 0.618

*Data expressed as mean ± SD, or number (percentage)

**Table 5:** Comparison of demographic characteristics between subgroups

Subgroup	Subgroup C1(*n* = 94)	Subgroup C2(*n* = 114)	Subgroup D1(*n* = 55)	Subgroup D2(*n* = 53)	*P*
Years of infertility	3.44 ± 2.58	3.12 ± 2.19	3.05 ± 2.22	4.53 ± 5.32	>0.05
Age (years)	33.62 ± 4.44	34.32 ± 4.05	32.95 ± 5.37	34.64 ± 4.35	>0.05
bFSH/bLH	1.77 ± 1.05	2.09 ± 1.90	1.91 ± 1.27	1.80 ± 1.38	>0.05

*Subgroup C1, Subgroup C2 basic data *p* values were: 0.344, 0.237, 0.140

*Subgroup D1, Subgroup D2 basic data *p* values were: 0.066, 0.075, 0.683

*Data expressed as mean ± SD, or number (percentage)

As shown in Table 6, the Gonadotropin dose was significantly lower (2200.34 ± 915.54 IU vs. 2571.68 ± 905.58 IU, *p* < 0.001) in Group 1 than in Group 2. However, the duration of ovarian stimulation, number of oocytes retrieved, and the number of available embryos were significantly higher(9.59 ± 2.12 vs. 9.01 ± 2.67, *p* = 0.036;8.17 ± 4.10 vs. 7.07 ± 4.05, *p* = 0.017;2.96 ± 2.03 vs. 2.52 ± 1.62, *p* = 0.036, respectively) in Group 1 than in Group 2. On the other hand, the duration of Cetrorelix use did not differ significantly between the groups. Moreover, fertilized oocytes and the number of superior-quality embryos did not differ significantly between the two groups.

**Table 6:** Comparison of laboratory data between the two groups

Groups	Group1 (*n* = 149)	Group2 (*n* = 167)	*P*
Cetrorelix usage days	3.44 ± 1.52	3.72 ± 1.71	0.115
Gn usage days	9.59 ± 2.12	9.01 ± 2.67	0.036
Gn total	2200.34 ± 915.54	2571.68 ± 905.58	0.000
retrieved oocytes	8.17 ± 4.10	7.07 ± 4.05	0.017
fertilized oocytes	4.89 ± 3.06	4.47 ± 3.00	0.220
available embryos	2.96 ± 2.03	2.52 ± 1.62	0.036
superior-quality embryos	0.80 ± 1.15	0.73 ± 0.97	0.568

*Gn* Gonadotropin

*Data expressed as mean ± SD, or number (percentage)

As shown in Table 7, Gonadotropin consumption was significantly lower (2201.40 ± 896.53 IU vs. 2498.29 ± 811.80 IU, 2197.62 ± 973.51 IU vs. 2628.67 ± 972.67 IU, *p* < 0.05) in subgroup A1/B1 than in subgroup A2/B2. Furthermore, the number of retrieved oocytes, fertilized oocytes, available embryos, and the number of superior-quality embryos did not differ significantly between subgroups A1/B1 and A2/B2.

**Table 7:** Comparison of laboratory data between subgroups

Subgroup	Subgroup A1(*n* = 107)	Subgroup A2(*n* = 73)	Subgroup B1(*n* = 42)	Subgroup B2(*n* = 94)	*P*
Cetrorelix usage days	3.52 ± 1.57	3.68 ± 1.68	3.21 ± 1.39	3.76 ± 1.73	>0.05
Gn usage days	9.71 ± 1.81	9.52 ± 2.83	9.29 ± 2.76	8.62 ± 2.51	>0.05
Gn total	2201.40 ± 896.53	2498.29 ± 811.80	2197.62 ± 973.51	2628.67 ± 972.67	<0.05
retrieved oocytes	8.37 ± 4.21	9.07 ± 3.95	5.88 ± 3.57	6.06 ± 3.63	>0.05
fertilized oocytes	5.38 ± 3.12	5.16 ± 3.31	3.64 ± 2.51	3.94 ± 2.64	>0.05
available embryos	2.85 ± 1.81	3.21 ± 2.24	2.33 ± 1.20	2.27 ± 1.42	>0.05
superior-quality embryos	0.86 ± 1.27	0.73 ± 0.93	0.64 ± 0.76	0.73 ± 1.00	>0.05

*Gn* Gonadotropin

*Subgroup A1, Subgroup A2 experimental data *P* values were:0.510, 0.584, 0.022, 0.253, 0.653, 0.259, 0.443

*Subgroup B1, Subgroup B2 experimental data *P* values were:0.077, 0.166, 0.018, 0.786, 0.544, 0.790, 0.598

*Data expressed as mean ± SD, or number (percentage)

As shown in Table 8, Gonadotropin consumption was significantly lower (2073.14 ± 934.23 IU vs. 2452.26 ± 896.32 IU, 2417.73 ± 847.28 IU vs. 2828.54 ± 879.56 IU, *p* < 0.05) in subgroup C1/D1 than in subgroup C2/D2. However, the number of retrieved oocytes was significantly higher (8.24 ± 4.04 vs. 6.83 ± 3.92, *p* = 0.012) in subgroup C1 than in subgroup C2.

**Table 8:** Comparison of laboratory data between subgroups

Subgroup	Subgroup C1(*n* = 94)	Subgroup C2(*n* = 114)	Subgroup D1(*n* = 55)	Subgroup D2(*n* = 53)	*P*
Cetrorelix usage days	3.35 ± 1.50	3.63 ± 1.60	3.58 ± 1.55	3.92 ± 1.91	>0.05
Gn usage days	9.28 ± 1.79	8.86 ± 2.93	10.13 ± 2.52	9.34 ± 2.06	>0.05
Gn total	2073.14 ± 934.23	2452.26 ± 896.32	2417.73 ± 847.28	2828.54 ± 879.56	<0.05
retrieved oocytes	8.24 ± 4.04	6.83 ± 3.92	8.05 ± 4.22	7.58 ± 4.31	C<0.05 D>0.05
fertilized oocytes	5.00 ± 2.94	4.35 ± 2.83	4.71 ± 3.26	4.74 ± 3.36	>0.05
available embryos	3.02 ± 2.04	2.61 ± 1.65	2.85 ± 2.03	2.34 ± 1.56	>0.05
superior-quality embryos	0.79 ± 0.98	0.70 ± 0.94	0.82 ± 1.40	0.79 ± 1.03	>0.05

*Gn* Gonadotropin

*Subgroup C1, Subgroup C2 experimental data *P* values were:0.198, 0.229, 0.003, 0.012, 0.107, 0.106, 0.523

*Subgroup D1, Subgroup D2 experimental data *P* values were:0.307, 0.788, 0.015, 0.568, 0.967, 0.143, 0.914

*Data expressed as mean ± SD, or number (percentage)

As shown in Table 9, there were 316 fresh embryo transfer cycles: *n* = 149 in Group 1 and *n* = 167 in Group 2. The clinical pregnancy rate (36.2 vs. 37.1%) and live birth rate (30.20 vs. 31.74%) were slightly higher in Group 2 than in Group 1. However, the differences were not statistically significant. The incidence of OHSS was not analyzed because some of the patients did not receive fresh embryo transfer to avoid OHSS.

**Table 9:** Comparison of clinical data between the two groups

Groups	Group1 (*n* = 149)	Group2 (*n* = 167)	*P*
clinical pregnancy rate	36.20%	37.10%	0.871
live production rate	30.20%	31.74%	0.768

*Data expressed as mean ± SD, or number (percentage)

As shown in Table 10, in the four subgroups based on age, the clinical pregnancy rate was slightly higher in subgroup A1 than in subgroup A2 (44.86 vs. 38.36%). However, the difference was not statistically significant. On the contrary, the live birth rate was slightly lower in subgroup A1 than in subgroup A2 (35.51 vs. 35.62%). However, the difference was also not statistically significant. However, for patients aged above 35 years, the clinical pregnancy rate (21.43 vs. 37.23%) and live birth rate (16.67 vs. 28.72%) were slightly higher in subgroup B2 than in subgroup B1. However, the differences were not statistically significant.

**Table 10:** Comparison of clinical data between subgroups

Subgroup	Subgroup A1(*n* = 107)	Subgroup A2(*n* = 73)	Subgroup B1(*n* = 42)	Subgroup B2(*n* = 94)	*P*
clinical pregnancy rate	44.86%	38.36%	21.43%	37.23%	>0.05
live production rate	35.51%	35.62%	16.67%	28.72%	>0.05

*Subgroup A1, Subgroup A2 pregnancy outcome *P* values were:0.386, 0.989

*Subgroup B1, Subgroup B2 pregnancy outcome *P* values were:0.069, 0.134

*Data expressed as mean ± SD, or number (percentage)

As shown in Table 11, there were 208 fresh embryo transfer cycles in the group with a BMI below 25: 94 in subgroup C1 and 114 in subgroup C2. The clinical pregnancy rate (34.04 vs. 43.86%) and live birth rate (27.66 vs. 35.96%) were slightly lower in subgroup C1 than in subgroup C2. However, the differences were not statistically significant. In contrast, in the group with a BMI greater than or equal to 25, the clinical pregnancy rate (45.45 vs. 24.53%, *P* = 0.023) and live birth rate (34.55 vs. 22.64%) were slightly higher in subgroup D1 than in subgroup D2. However, the difference in live birth rates was not statistically significant.

**Table 11:** Comparison of clinical data between subgroups

Subgroup	Subgroup C1(*n* = 94)	Subgroup C2(*n* = 114)	Subgroup D1(*n* = 55)	Subgroup D2(*n* = 53)	*P*
clinical pregnancy rate	34.04%	43.86%	45.45%	24.53%	C>0.05 D<0.05
live production rate	27.66%	35.96%	34.55%	22.64%	>0.05

*Subgroup C1, Subgroup C2 pregnancy outcome *P* values were:0.149, 0.202

*Subgroup D1, Subgroup D2 pregnancy outcome *P* values were:0.023, 0.172

*Data expressed as mean ± SD, or number (percentage)

## Discussion

In recent years, GnRH antagonists have been increasingly used in IVF / ICSI ovulation induction therapy. GnRH antagonists do not result in pituitary down-regulation associated with the GnRH agonist regimen. In addition, the GnRH antagonist regimen is advantageous, as it has milder stimulation and a shorter duration of administration [9]. The pregnancy rate achieved with GnRH-ant protocol is considered lower than with GnRH-agonist protocol. However, this is controversial. The lower pregnancy rate with GnRH-ant protocol is associated with impaired endometrial receptivity.

Many studies have reported the patient’s age and BMI to be among the factors that influence outcomes in IVF-ET with the antagonist regimen [10, 11]. Doctors in reproductive health services have opted to lower the GnRH-ant dose to achieve satisfactory pregnancy outcomes. However, there is no clear guide on the dosage of antagonists. Currently, 0.25 mg of Cetrorelix per day, started on day 6 of ovarian stimulation, is the standard GnRH-ant protocol to maintain the LH level within a safe range. However, some studies have reported that lower GnRH-ant doses of 0.125–0.2 mg per day are also effective [12, 13]. Therefore, this study compared IVF-ET outcomes in patients receiving 0.125 mg and 0.25 mg Cetrorelix daily to determine the appropriate dose.

First, we found no differences in terms of fertilized oocytes and superior-quality embryos between the two groups. However, there were significant differences in the number of retrieved oocytes and available embryos between the two groups. The A dosage of 0.125 mg per day was higher than 0.25 mg per day. In addition, the duration of stimulation with GnRH-Ant was significantly shorter in group 1 than in group 2. Many studies have reported that GnRH-Ant has adverse effects on endometrial receptivity [14, 15]. In addition, some previous studies also reported that increasing doses of GnRH antagonists led to an increase in uterine natural killer (UNK) cells and inflammatory factors such as perforin and tumor necrosis factorα (TNFα) [16, 17]. Furthermore, prolonged ovarian stimulation was associated with a decreased superior-quality embryos and live birth rates [18–20]. In this present study, the clinical pregnancy rate and live birth rate were lower in Group 1 than Group 2, although this difference was not statistically significant. These findings contrast previously reported studies [21]. The retrospective nature of this study and the small sample size might explain this finding. Therefore, it is essential to carry out a multicenter randomized controlled study to validate these findings.

Second, there was no difference in the number of retrieved oocytes, fertilized oocytes, available embryos, the number of superior-quality embryos, the clinical pregnancy rate and live birth rate among the four subgroups based on age. These findings suggest that age does not significantly affect the GnRH-ant dose.

Thirdly, for patients with a BMI < 25, the number of oocytes retrieved in the 0.125 mg group (subgroup C1) was significantly higher than in the 0.25 mg group (subgroup C2). In addition, for patients with a BMI of 25 or more, the clinical pregnancy rate in the 0.125 mg subgroup (subgroup D1) was significantly higher than in the 0.25 subgroup (subgroup D2). This finding suggests that BMI may influence the GnRH-ant dose. According to Engel et al., a patient’s body weight does not affect the plasma concentration of Cetrorelix, and there is no need to modify the dose during COS [22]. In contrast, a few studies have suggested the need to reduce the GnRH-ant dose in slim patients [23].

## Conclusion

In conclusion, a lower GnRH-ant dose of 0.125 mg per day has clinical advantages over daily doses of GnRH-ant 0.25 mg per day in patients with a BMI greater than or equal to 25. However, age had no significant influence on the GnRH-ant dose. Therefore, by considering the effect of GnRH-ant on the endometrium and the economic aspects, a daily dose of 0.125 mg GnRH-ant is recommended. The results of this retrospective analysis need to be validated in a large sample size RCT.

## Data Availability

The datasets used and/or analyzed in this study will be availed by the corresponding author upon a reasonable request.
